# The application of 3D-printed oral stents in intensity-modulated radiotherapy for oropharyngeal cancer and their dosimetric effect on organs at risk

**DOI:** 10.1186/s40001-023-01333-x

**Published:** 2023-09-22

**Authors:** Jungang Ma, Zhuo Chen, Shuixia Liu, Wei Hu, Kunpu Su, Rong He, Peng Zhou, He Xiao, Jia Ju, Qianying Hou, Yinying Zhou, Bin Wang

**Affiliations:** 1 Department of Oncology, Daping Hospital, Army Medical University, 10 Changjiang Branch Road, Chongqing, 400042 China; 2 Department of Critical Care MedicineDaping Hospital, Army Medical University, Chongqing, 400042 China; 3 Department of Oncology, The Seventh People’s Hospital of Chongqing (Affiliated Central Hospital of Chongqing University of Technology), Lijiatuo Street, Chongqing, 400054 China

**Keywords:** Oropharyngeal cancer, 3D printing, Oral stent, Dosimetry, Organs at risk

## Abstract

**Background:**

This study investigates the accuracy of 3D-printed dental stents in intensity-modulated radiotherapy (IMRT) for oropharyngeal cancer (OPC) and their dosimetric effects on normal tissues.

**Methods:**

We selected 60 patients with OPC who underwent IMRT in the Department of Oncology, Special Medical Center of Army Medical University. These patients were randomly assigned into 3D-printed oral stent, simple glass bottle, and nonstent groups (20 patients/group). The positioning error was analyzed with the onboard imaging system once a week after 5 fractions of IMRT. The conformity index (CI), homogeneity index (HI), radiation dose of organs at risk (OARs), and oral mucosal reaction were compared among the three groups.

**Results:**

No significant difference was observed in the conformity and uniformity of the target dose and the dose received by the spinal cord, larynx, and bilateral parotid glands among the three groups (*P* > 0.05). Meanwhile, the dose received by the upper cheek, hard palate, and soft palate of patients was significantly lower in the 3D-printed oral stent group than in the nonstent group (*P* < 0.05) but insignificantly different between the 3D-printed oral stent and simple glass bottle groups (*P* > 0.05). When compared with the nonstent group, the simple glass bottle group showed a markedly lower dose received by the upper cheek (*P* < 0.05) and an insignificantly different dose received by the hard palate and soft palate (*P* > 0.05). According to Common Terminology Criteria for Adverse Events v.5.0, the adverse response rate of the hard palate mucosa was lower in the 3D-printed oral stent group than in the simple glass bottle and nonstent groups (*P* < 0.05).

**Conclusions:**

For OPC patients undergoing IMRT, the application of 3D-printed oral stents can significantly reduce the exposure dose of the upper cheek and hard palate and decrease the occurrence of adverse events such as oral mucositis although it cannot affect the positioning error.

## Background

Oropharyngeal cancer (OPC) is a malignancy occurring in the palatine tonsil, soft palate, tongue root, pharyngeal wall, and surrounding epiglottis, accounting for around 1.3% of malignancies in the whole body [1, 2]. OPC is characterized by high invasion, rapid progression, and high potential of distant metastasis to the lungs, liver, and bones [3, 4]. Currently, it is extensively recognized that the incidence of OPC is predominantly attributable to poor oral hygiene, the long-term friction of the buccal mucosa, the stimulation of tobacco, alcohol, and betel nut, the susceptibility of the body, genetics, and nutritional metabolic disorders [2]. As one of the main treatments for oral cancer, radiotherapy has been used in various clinical settings, such as postsurgical subclinical irradiation and radical radiotherapy for inoperable patients. Of course, radiotherapy for OPC also exhibits limitations, mainly including difficult positioning, uneven doses, limited exposure due to the complex oral anatomy, and the high incidence of local adverse events in the oral cavity [5, 6], which calls for the needs to further elevate the accuracy of radiotherapy for oral cancer and reduce radiation damage.

Intensity-modulated radiation therapy (IMRT) has been widely utilized in clinical practice as a result of the rapid development of radiotherapy equipment. More importantly, this technology has been extensively applied for the treatment of head and neck tumors because of its characteristics of accurate target positioning, optimized target dose distribution, and maximum normal tissue protection, combined with its dosimetric advantages. Nevertheless, IMRT still has certain limitations, including oropharyngeal mucosal damage and local adverse reactions, although its advances have improved the accuracy and efficacy of radiotherapy in OPC treatment. Reportedly, individualized oral stents designed based on the oral structure of patients can effectively separate normal tissues from the tumor target and protect oral buccal mucosa, tongue, hard palate, soft palate, posterior pharyngeal wall, and other normal tissues to reduce the dose of radiation and the incidence of toxic effects. Oral stents were first used clinically for radiotherapy in 1965 [7] and have recently been applied in radiotherapy for oral, oropharyngeal, and nasopharyngeal cancers [8], with certain clinical effectiveness. A prior study reported that the use of oral stents in radiotherapy for head and neck tumors markedly diminished the dose of the tongue and the incidence of tongue mucositis and prevented taste damage [9]. Several studies have confirmed that individualized oral stents can decrease the dose and volume of radiation of the tongue, oral mucosa, and upper and lower gingival mucosa without affecting the dose of the target area, protect parotid glands, mandible, middle ear, and other normal tissues, and diminish the occurrence of taste damage [10, 11]. Accordingly, oral stents are worthy of clinical promotion. In recent years, oral stents have been extensively utilized in radiotherapy for nasopharyngeal cancer by the tumor radiotherapy team of Sun Yat-sen University in China. Furthermore, the materials used for stents have been evolving [12]. Specifically, oral stents containing gold compounds, pure titanium, amalgam, and artificial materials have been developed by different groups [13]. For instance, Zheng et al. [14] developed a model with anhydrite and mixed the self-setting denture base with resin and plastic occlusal pads for the treatment of nasopharyngeal cancer, which showed high efficacy. Of note, the constant improvement of radiotherapy for oral cancer has spurred increasingly high requirements for the manufacture and design of oral stents. In addition, sound manufacturing processes can elevate the quality of oral stents and ensure stent stability during treatments [15]. For example, Liu et al. [16] observed that the isocenter of three metal points on individualized oral stents prepared with methacrylic resin had small three-dimensional (3D) vector displacement in the X, Y, and Z axes, which improved the efficacy of radiotherapy in the treatment of nasopharyngeal cancer. In addition, Chen et al. [17] analyzed and summarized the positioning error of 21 patients with head and neck tumors who used dental mouthpieces and corks as oral stents in radiotherapy and found that the positioning error in the cork group was significantly higher than that of the mouthpiece group. At present, oral stents have gained widespread adoption in radiotherapy for patients with head and neck tumors in developed countries. However, simple equipment, such as glass bottles, corks, and syringes are still utilized as oral stents in China [18]. Although these types of equipment are readily produced and less costly with certain correction effects, they cannot be used alone and then are of limited clinical application due to their large displacement, poor reproducibility, and low safety in the clinic. Consequently, it is necessary for improving the accuracy and efficacy of oral stents to find better materials.

In this research, we compared the positioning errors and organ doses among OPC patients with stent-free radiotherapy, oral stents constructed with 3D printing technology, and simple glass bottles in the mouth, thereby providing a reference for the development or selection of individualized radiation protection equipment for OPC patients undergoing radiotherapy in the future.

## Methods

### Patient information

This study enrolled 60 OPC patients (31 males and 29 females; aged 38–68 years with a mean age of 51.3 years) who underwent IMRT at the Army Medical Center from August 2010 to December 2018, including. According to the 8th edition of the tumor–node–metastasis staging criteria for OPC published by the American Joint Committee on Cancer, these patients included 11 cases of T1N0M0 stage, 15 cases of T2aN0M0 stage, 10 cases of T1N1M0 stage, 12 cases of T2aN1M0 stage, 3 cases of T2bN1M0 stage, and 4 cases of T2bN2M0 stage, and 5 cases of T3N0M0 stage. All patients were pathologically confirmed as squamous cell carcinoma, including 22 cases of tonsil cancer, 18 cases of soft palate cancer, and 20 cases of tongue root cancer. The inclusion criteria of patients were as follows: patients with negative margins; patients without psychiatric disorders and other related diseases; patients with the ability to tolerate chemoradiotherapy; patients with acceptable oral dental status; patients without severely restricted mouth opening; patients with the ability to bite a stent or hold a bottle in mouth. The patients were randomly classified into three groups: 3D-printed oral stent (patients with a 3D-printed oral stent in mouth), simple glass bottle (patients with a simple glass bottle in mouth), and control (patients without a stent, nonstent) groups. Except for age, baseline characteristics, such as physical fitness score, tumor type, and clinical stage, were not statistically significantly different among the three groups (*P* > 0.05) (Table 1).

**Table 1:** Patient characteristics

	Stent	Bottle	Control	*χ*^2^	*P* value
Sex					
Male	11	11	9	0.534	0.766
Female	9	9	11		
Age					
≤ 60	17	16	17	25.417	< 0.001
> 60	3	4	3		
Tumor site					
Tonsil	8	8	6	1.397	0.845
Soft palate	5	7	6		
Tongue root	7	5	8		
TNM stage					
TIN0M0	3	4	4	2.811^†^	1.000
T2aN0M0	6	4	5		
T1N1M0	3	4	3		
T2aN1M0	4	4	4		
T2bN1M0	1	1	1		
T2bN2M0	2	1	1		
T3N0M0	1	2	2		

^†^Fisher’s exact probability; χ^2^, represents the statistic of the result of the χ^2^ test; *P* value, is a measure of the statistical significance of the χ^2^ statistic

### Main equipment

In this study, we used a Synergy Linear Accelerator, a MOSAIQ system, a MONACO treatment planning system (Elekta AB, Stockholm, Sweden), a computed tomography (CT) simulator (Philips, Amsterdam, the Netherlands), a movable laser light system (LAP, Germany), and a Stratasys F123 3D printer (Stratasys, Rehovot, Israel).

### 3D-printed oral stents, simple glass bottle, and nonstent

**Fig. 1:**
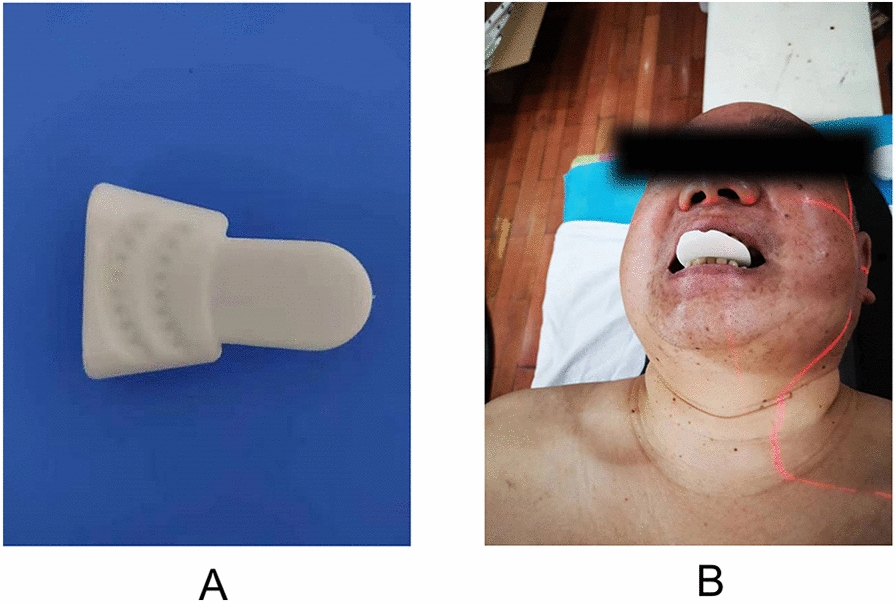
The 3D-printed oral stent (**A**) and schematic diagram of the patient (**B**)

The occlusion degree of the upper and lower incisor teeth of each patient was set at approximately 2.5 cm based on the principle of individual difference and patient comfort. Specifically, the softened impression paste was placed into the mouth of patients, and then patients were instructed to bite the paste into a synthetic shape. After 5 min, the model was taken out of the mouth, rinsed with water, and scanned on a large-aperture positioning CT simulator with a slice thickness of 1.25 mm and a slice spacing of 0.625 mm. Subsequently, the CT images were imported into Mimics 10.1 software to discard redundant data, followed by the reconstruction of a 3D model. According to the oral condition of patients in MEDCAD, the ventilation channel was outlined in the module. After the 3D model was obtained and further optimized, an individualized oral stent was printed with the set printing speed and slice height by inputting the information into a 3D printer. Figure 1 shows the 3D-printed oral stent and schematic diagram of the patient. The main material of the 3D-printed oral stent used in this study was polylactic acid (PLA) which is a popular 3D printing filament derived from renewable sources such as cornstarch or sugarcane. It is biodegradable, relatively inexpensive, and offers moderate strength for oral stent applications [19, 20].

Patients in the glass group had a simple glass bottle placed in their mouth as a dental stent, while the control group did not use any stent. All the Patients lay in a supine position. Appropriately angled head and shoulder cushions were placed on the head and shoulders of patients and fixed with S-shaped thermoplastic masks.

### Radiotherapy positioning and target area delineation

CT scanning was performed with a slice thickness of 3 mm, a slice spacing of 3 mm, and a reconstruction matrix of 512 × 512 and reached up to the top of the skull and down to 5 cm below the clavicle head. A cross was created with a 3D laser light on the left, middle, and right of the mask. The lead wire was pasted to the mask as a CT imaging mark, followed by CT scanning. After the scanning, the oral stent or simple glass bottle was removed, and the mask was worn. Next, CT scanning was performed again with the same parameters according to the same three crosses. All of the obtained image data were transported to the MIM image for fusion and uploaded to the planning system Monaco for target area delineation and planning design. As per ICRU 50 and ICRU 62 reports, the radiotherapist outlined the radiotherapy target area and normal tissues including the spinal cord, larynx, bilateral parotid gland, upper cheek, and soft palate. Thereafter, the approved plan was imported into the Elekta linear accelerator system. All patients underwent IMRT, during which all irradiation fields were used with standardized operations. The prescribed dose was 60 Gy/30 fractions. The same organ-at-risk (OAR) dose constraint parameters were utilized for all plans, and all IMRT plans were completed by an experienced physician.

### Registration image acquisition and plan quality evaluation

After the radiotherapist outlined the target area, the physician comprehensively evaluated the different dosimetry data in the plan based on the isodose curve distribution and dose volume histogram on the MONACO5.11.01 planning system. cone beam CT (CBCT) scanning was conducted for all patients. The CBCT images were compared with the localized images. The frame including the tumor target and nearby fixed bone structures was registered with the bone registration method. The XVI image registration software was used for registration to obtain the linearity and rotation errors of X, Y, and Z axes. The X-axis referred to the left–right direction, where the left was positive and the right was negative. The Y axis represented the head–feet direction, where the head was positive and the foot was negative.

### Target dosimetry identification

Target uniformity and conformity were assessed with planning target volume (PTV) dose coverage (coverage = V95 − gross tumor volume [GTV]/virtual GTV [VGTV]), heterogeneity index (HI), conformity index (CI), and 95% volume of the target area (D95). In detail, V95–GTV represented the volume of GTV included in 95% of the prescribed dose line, and VGTV referred to the volume of GTV. D95–GTV represented the maximum dose of radiation received by 95% of the GTV volume, and Dmax–GTV represented the maximum dose of GTV. The CI was calculated with the following formula: CI = VRX^2^/(TV · VRI), in which VRX was the volume of the target area covered by the prescribed dose, TV was defined as the volume of the target, and VRI represented the volume enclosed by the isodose line of the prescribed dose [21]. The CI value ranged from 0 to 1. The higher CI value was associated with better conformity. The HI was calculated with the following formula: HI = D5%/D95%, where D5% referred to the dose received by the hottest 5% target, and D95% was the minimum dose received by 95% volume of the target [22]. The higher HI value indicated worse dose distribution.

### Oral mucosal reaction

Patients were observed for the condition of the mucosa of the upper cheek, soft palate, and hard palate in the mouth before radiotherapy, at the end of 2, 4, and 6 weeks of radiotherapy, and 1 and 3 months after radiotherapy. Afterward, oral mucosa radiological responses were evaluated with the Common Terminology Criteria for Adverse Events (CTCAE) v.5.0 published by the US Department of Health and Human Services in November 2017.

### Statistical analysis

SPSS 22.0 statistical software was used for all statistical analyses. The clinical baseline characteristics of the three groups were compared with the Chi-square test or Fisher exact probability method. The measurement data were expressed as mean ± standard deviation. The one-way analysis of variance was utilized for the analysis of the differences in positioning errors, dosimetry parameters, and OARs and pairwise comparisons. The Bonferroni method was used for multiple comparison corrections. The Kruskal–Wallis rank sum test was used for comparing taste damage and mucosal reaction among the three groups. All analyses were two-sided tests, and *P* < 0.05 was considered statistically significant.

## Results

### Positioning error analysis

**Fig. 2:**
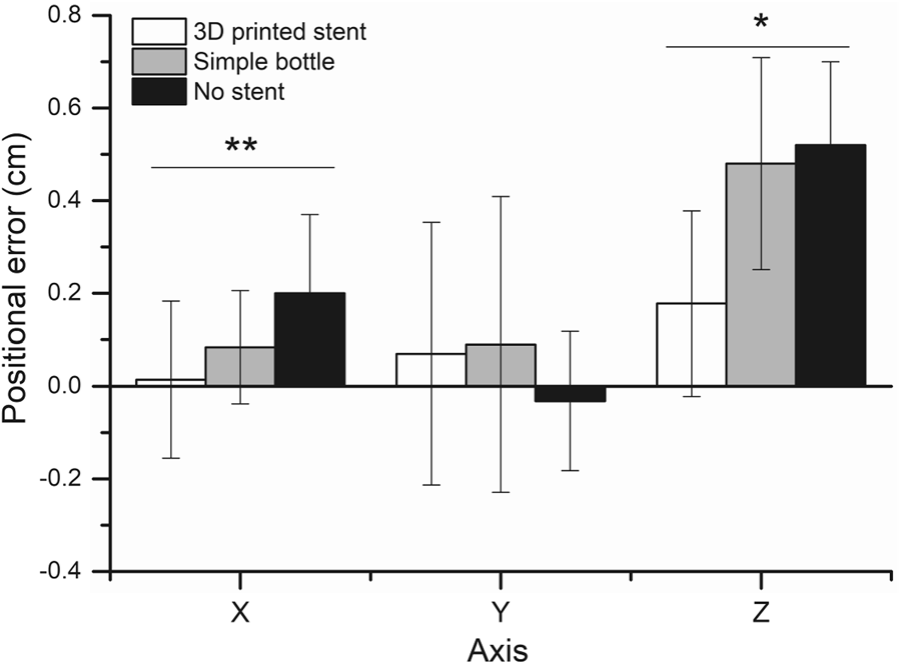
Comparisons of positioning errors among the three groups: *P < 0.05, **P < 0.01

All patients underwent CBCT scanning and positioning corrections. After each positioning correction, the positioning error in the 3D direction was obtained, where the X-axis referred to the left–right directions (the left was positive, and the right was negative), the Y axis referred to the head–foot direction (the head was positive, and the foot was negative), and the Z axis represented the forward–backward direction (the forward was positive, and the backward was negative). The mean ± standard deviation in X axis (left and right), Y axis (up-down), and Z axis (front–back) directions were 0.014 ± 0.169, 0.07 ± 0.283, and 0.178 ± 0.2 cm in the 3D-printed oral stent group, respectively. The mean ± standard deviation of the simple glass bottle group was 0.084 ± 0.122 cm on the X axis, 0.09 ± 0.319 cm on the Y axis, and 0.48 ± 0.229 cm on the Z axis. The nonstent group had the mean ± standard deviation of 0.2 ± 0.17 cm on the X axis, − 0.032 ± 0.15 cm on the Y axis, and 0.52 ± 0.18 cm on the Z axis (Table 2, Fig. 2). Significant differences were found in the positioning error of X and Z axes among these three groups (Fig. 1).

**Table 2:** Analysis of IMRT Posture Errors (mean ± standard deviation, cm)

Group	*n*	X	Y	Z
3D-printed stent	20	0.014 ± 0.169	0.07 ± 0.283	0.178 ± 0.2
Simple glass bottle	20	0.084 ± 0.122	0.09 ± 0.319	0.48 ± 0.229
Non-stent	20	0.2 ± 0.17	− 0.032 ± 0.15	0.52 ± 0.18
*P* value		0.003	0.100	0.040

### Target dose distribution analysis

The target dose distribution of IMRT in 60 patients is displayed in Table 3. There was no marked difference in the median dose of Dmax, HI, and CI between the targets of the three groups. These results indicated that the dose distribution of the target volume for IMRT in the three groups could meet the requirements of clinical treatment.

**Table 3:** The mean value of PTV60

PTV_60_	Stent	Bottle	Control	*F* (df_1_ = 2, df_2_ = 57)	*P* value
D95(Gy)	61.56 ± 1.169	62.27 ± 1.283	62.78 ± 1.2	2.084	0.134
HI	1.21 ± 0.69	1.18 ± 0.72	1.11 ± 0.64	2.187	0.122
CI	0..77 ± 0.18	0.68 ± 0.12	0.64 ± 0.19	0.457	0.635
Coverage	1.00 ± 0.01	1.00 ± 0.03	1.00 ± 0.04	2.585	0.084

F, represents the F-statistic used in ANOVA to compare variances between groups; *P* value, associated with the F-statistic measures the statistical significance of the observed differences

### Comparisons of OAR dose distribution

**Fig. 3:**
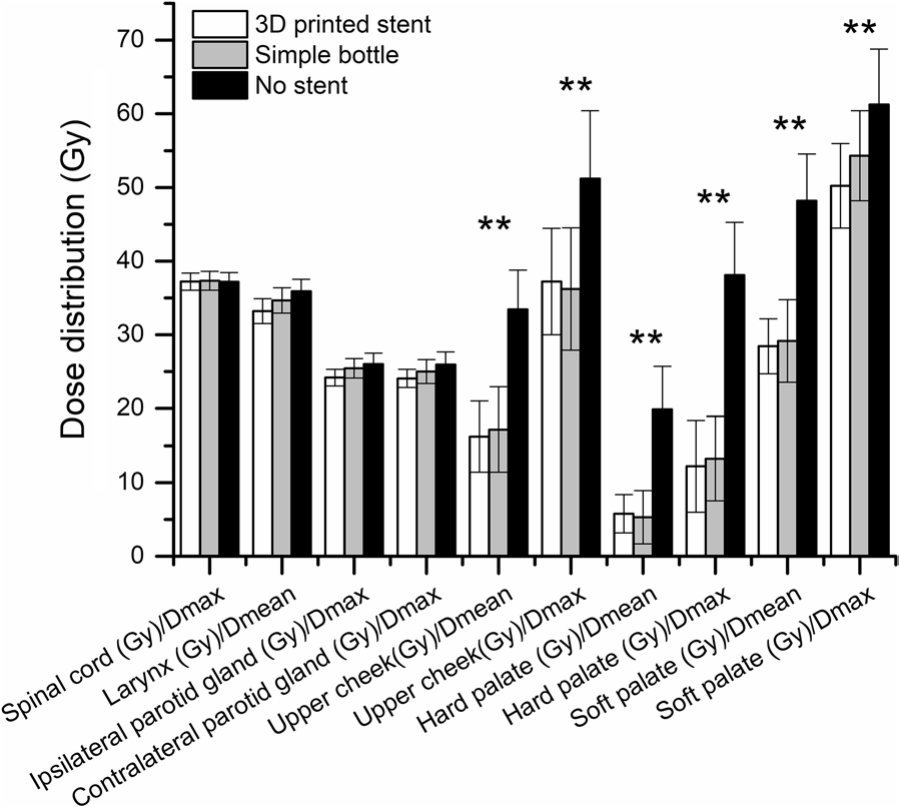
Comparisons of OAR dose distribution: **P < 0.01

The dose distribution of the spinal cord, larynx, and bilateral parotid glands was not statistically significant in patients from the three groups. However, the three groups exhibited a substantial difference in the Dmax and Dmean of the upper cheek, hard palate, and soft palate (Table 4, Fig. 2). A pairwise comparison demonstrated that the Dmax and Dmean of the upper cheek, hard palate, and soft palate were significantly lower in the 3D-printed oral stent group than in the nonstent group (adjusted *P* < 0.01) but insignificantly different between the 3D-printed oral stent and simple glass bottle groups (adjusted *P* > 0.05). In addition, as compared to the nonstent group, the simple glass bottle group showed lower Dmax and Dmean of the upper cheek (adjusted *P* < 0.05), accompanied by no significant change in the Dmax and Dmean of hard and soft palates (*P* > 0.05) (Fig. 3) which may be explained by the shape and material of the hard and soft palates.

**Table 4:** Comparisons of dose-volume parameters of major organs at risk for IMRT in the three groups

Endangered organ	Stent	Bottle	Control	*F* (df_1_ = 2, df_2_ = 57)	*P* value
Spinal cord (Gy)/D_max_	37.22 ± 1.169	37.32 ± 1.283	37.18 ± 1.26	2.118	0.130
Larynx (Gy)/D_mean_	33.21 ± 1.69	34.68 ± 1.72	35.91 ± 1.64	2.199	0.120
Ipsilateral parotid gland (Gy)/D_max_	24.19 ± 1.12	25.46 ± 1.32	26.03 ± 1.49	0.432	0.651
Contralateral parotid gland (Gy)/D_max_	24.08 ± 1.23	25.02 ± 1.62	25.95 ± 1.74	2.568	0.086
Upper cheek (Gy)/D_mean_	16.22 ± 4.82	17.18 ± 5.77	33.45 ± 5.34	6.418	0.003
Upper cheek (Gy)/D_max_	37.22 ± 7.23	36.22 ± 8.34	51.22 ± 9.19	6.091	0.004
Hard palate (Gy)/D_mean_	5.77 ± 2.61	5.31 ± 3.61	19.88 ± 5.84	8.926	< 0.001
Hard palate (Gy)/D_max_	12.21 ± 6.23	13.24 ± 5.72	38.11 ± 7.16	10.365	< 0.001
Soft palate (Gy)/D_mean_	28.44 ± 3.72	29.17 ± 5.61	48.21 ± 6.35	12.672	< 0.001
Soft palate (Gy)/D_max_	50.22 ± 5.74	54.32 ± 6.11	61.25 ± 7.54	5.798	0.005

### Oral mucosal reactions of patients

**Fig. 4:**
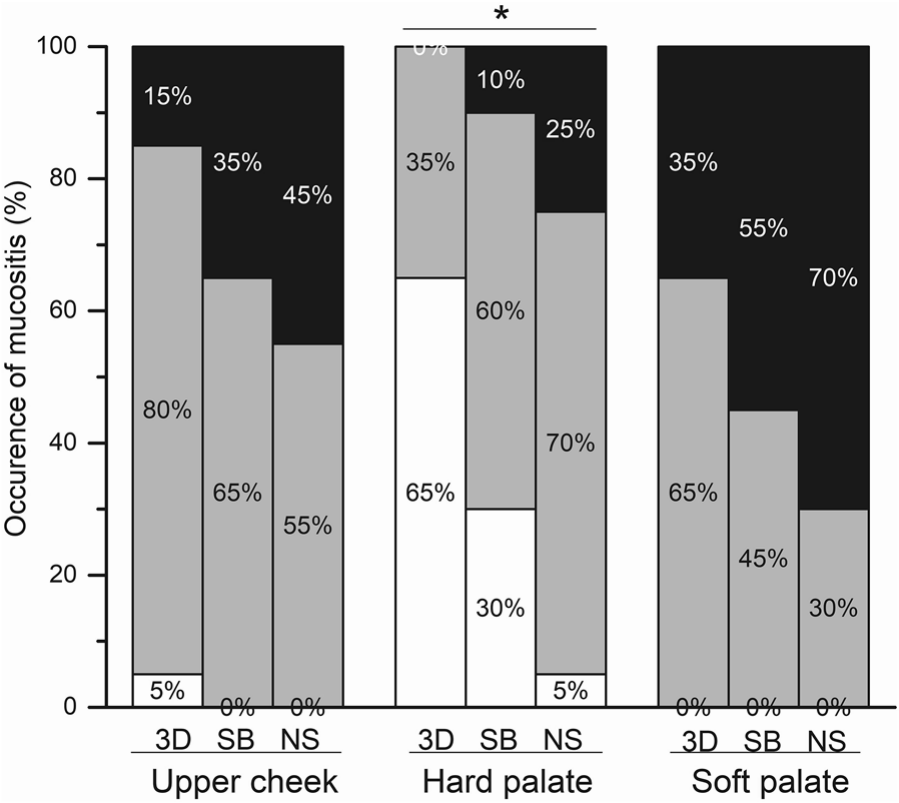
The incidence of mucositis in the three groups: 3D: stent; SB: bottle; NS: control. *P < 0.05

The incidence of radiation-induced oral mucositis was analyzed based on CTCAE v.5.0. As exhibited in Table 5, mucosal reactions mainly occurred in the upper cheek and soft palate, and almost all patients in the three groups suffered from radio-mucosal reactions in the upper cheek and soft palate. Moreover, no statistically significant difference was observed among the three groups in terms of the incidence of radiation-induced adverse events in the upper cheek and soft palate (*P* > 0.05). However, the 3D-printed oral stent group had a markedly lower radiological mucosal response rate at the hard palate than the simple glass bottle and nonstent groups (*P* < 0.05) (Fig. 4).

**Table 5:** Comparisons of the occurrence of mucositis in the oral mucosa among the three groups (CTCAE v.5.0)

Site	Stent			Bottle			Control			*P* value
	Grade 0	Grade 1–2	Grade 3	Grade 0	Grade 1–2	Grade 3	Grade 0	Grade 1–2	Grade3	
Upper cheek	1	16	3	0	13	7	0	11	9	*P* > 0.05
Hard palate	13	7	0	6	12	2	1	14	5	*P* < 0. 05
Soft palate	0	13	7	0	9	11	0	6	14	*P* > 0.05

## Discussion

Currently, there are various oral stents used to address mucosal damage and local adverse reactions caused by IMRT. Therefore, it is necessary to search for better materials to improve the accuracy and efficacy of oral trays. In this study, we compared the positioning errors and organ doses between patients receiving treatment without a tray, patients using an oral tray constructed with 3D printing technology, and patients using a simple glass bottle placed in the mouth. Our data unraveled that this individualized oral stent produced based on the oral structure of patients can be ideally adapted to the oral environment of patients and protect oral buccal mucosa, tongue, hard palate, soft palate, posterior pharyngeal wall, and other normal tissues.

Clinically, 3D printing technology has been widely used in surgery. This technology mainly uses continuous layer-by-layer printing and stratifies 3D mathematical model data to finally form a 3D solid stent, which can be used to simulate the surgical process for precise surgery. The 3D printing technology has many advantages, such as improving manufacturing accuracy, simplifying tedious production processes, saving cost and human resources, shortening production time, and achieving personalized production [23]. Currently, some new 3D printing materials are also widely used in stomatology, including metals, polymers, ceramics, and bioactive materials.

3D-printed oral stents produced from PLA were used in precision radiotherapy for OPC patients in the current study. PLA was selected as a material for the main piece because of its biodegradability, biocompatibility, and strength [19, 20]. PLA is now successfully used in radiotherapy for other cancers [24, 25], with resistance to radiation damage [26]. Kouji Katsura et al. have found that the material of the oral stent may affect the dose distribution for during external beam radiotherapy, the presence of dental alloys can cause an increase in mucosal doses due to backscatter radiation [27]. Another study also found that the utilization of a 3D-printed oral positioning radiotherapy stent proves to be a viable and consistent method, resulting in significant reductions of 42%, 21%, and 8.5% in the planning target volume and radiation doses administered to the hard palate, right parotid gland, and left parotid gland respectively [28]. Similarly, we also found that the 3D-printed oral stent group exhibited substantially lower doses in the upper cheek, hard palate, and soft palate than the nonstent group. The Dmax and Dmean of the upper cheek, hard palate, and soft palate were markedly lower in the 3D-printed oral stent group than in the simple glass bottle group. Increased sample size may contribute to improved effects. Meanwhile, the intensity-modulated dosimetry was also used to analyze the dose suitability and uniformity of the target area in the three groups, which demonstrated no significant difference. The dose distribution of important organs, including the spinal cord, larynx, and bilateral parotid gland, also showed no difference among the three groups, illustrating that 3D-printed oral stents are safe and reliable. We speculate that the physical reason that lower adverse for 3D-printed oral stent were as follows: Firstly, the 3D-printed oral stent is customized specifically for each patient, ensuring accurate positioning and stability during radiotherapy. Secondly, the use of a 3D-printed oral stent, made from materials such as PLA, reduces the presence of these alloys or materials, resulting in a decrease in backscatter radiation and lower doses to adjacent structures. In addition, the physical properties of the 3D-printed oral stent, such as its shape and composition, can provide a shielding effect.

In addition, our results illustrated that the lower mean ± standard deviations of the 3D-printed oral stent group in X axis (left and right), Y axis (up and down), and Z axis (front–back) directions, indicating that 3D-printed oral stent is more stable as oral stents than other equipment, such as simple glass bottle, and nonstent. Furthermore, according to the CTCAE v.5.0, the 3D-printed oral stent group displayed a lower adverse response rate in the hard palate mucosa than the simple glass bottle and nonstent groups, with a significant difference. Of course, we also observed no substantial difference in the dose received by the hard and soft palates among the three groups and a reduction in the dose received by the upper cheek in the simple glass bottle and nonstent groups, indicating that the application of simple glass bottles with mouth remains to be investigated.

## Conclusions

In summary, for OPC patients undergoing IMRT, the application of 3D-printed oral stents cannot significantly affect the positioning error but significantly reduces the exposure dose of the upper cheek and hard palate, thereby diminishing the incidence of adverse reactions, such as oral mucositis. However, we note that compared with advanced oral stents, such as methyl methacrylate resin, dental gum, and 3D printing, the materials used in this oral stent are not clinically used. Therefore, it is unclear whether there are any adverse effects. In addition, the number of cases enrolled in this study is relatively small. Consequently, our results are warranted to be further confirmed in future studies with large sample sizes.

## Data Availability

The datasets used and/or analyzed during the current study are available from the corresponding author on reasonable request.

## Abbreviations

3D: Three dimensional
IMRT: Intensity-modulated radiotherapy
OPC: Oropharyngeal cancer
CI: Conformity index
HI: Homogeneity index
OARs: Organs at risk
IMRT: Intensity-modulated radiation therapy
CT: Computed tomography
OAR: Organ at risk
CBCT: Cone beam CT
PTV: Planning target volume
GTV: Gross tumor volume
VGTV: Virtual GTV
CTCAE: Common terminology criteria for adverse events

## References

[1] Lorenzoni V, Chaturvedi AK, Vignat J, Laversanne M, Bray F, Vaccarella S (2022). The Current Burden of Oropharyngeal Cancer: a global assessment based on GLOBOCAN 2020. Cancer Epidemiol Biomarkers Prev.

[2] Huber MA, Tantiwongkosi B (2014). Oral and oropharyngeal cancer. Med Clin North Am.

[3] Ding J, Tu W, Hu H, Shi H, Kong Y (2017). Design of individualized oral radiotherapy stent based on 3D printing technique. Zhongguo Yi Liao Qi Xie Za Zhi.

[4] Geiger JL, Ku JA (2019). Postoperative treatment of oropharyngeal cancer in the era of human papillomavirus. Curr Treat Options Oncol.

[5] Wilke CT, Zaid M, Chung C, Fuller CD, Mohamed ASR, Skinner H, Phan J, Gunn GB, Morrison WH, Garden AS, Frank SJ, Rosenthal DI, Chambers MS (2017). Design and fabrication of a 3D-printed oral stent for head and neck radiotherapy from routine diagnostic imaging. 3D Print Med..

[6] Meng Z, Garcia MK, Hu C, Chiang J, Chambers M, Rosenthal DI, Peng H, Zhang Y, Zhao Q, Zhao G, Liu L, Spelman A, Palmer JL (2012). Randomized controlled trial of acupuncture for prevention of radiation-induced xerostomia among patients with nasopharyngeal carcinoma. Cancer.

[7] Santiago A (1965). An intraoral stent for the direction of radiation beam therapy. J Prosthet Dent.

[8] Doi H, Tanooka M, Ishida T, Moridera K, Ichimiya K, Tarutani K, Kitajima K, Fujiwara M, Kishimoto H, Kamikonya N (2017). Utility of intraoral stents in external beam radiotherapy for head and neck cancer. Rep Pract Oncol Radiother..

[9] Wang H, Wang C, Tung S, Dimmitt AW, Wong PF, Edson MA, Garden AS, Rosenthal DI, Fuller CD, Gunn GB, Takiar V, Wang XA, Luo D (2016). Improved setup and positioning accuracy using a three-point customized cushion/mask/bite-block immobilization system for stereotactic reirradiation of head and neck cancer. J Appl Clin Med Phys.

[10] Qin WJ, Luo W, Lin SR, Sun Y, Li FM, Liu XQ, Ma J, Lu TX (2007). Sparing normal oral tissues with individual dental stent in radiotherapy for primary nasopharyngeal carcinoma patients. Ai Zheng.

[11] Deshpande TS, Blanchard P, Wang L, Foote RL, Zhang X, Frank SJ (2018). Radiation-related alterations of taste function in patients with head and neck cancer: a systematic review. Curr Treat Options Oncol.

[12] Wang RR, Olmsted LW (1995). A direct method for fabricating tongue-shielding stent. J Prosthet Dent.

[13] Reitemeier B, Reitemeier G, Schmidt A, Schaal W, Blochberger P, Lehmann D, Herrmann T (2002). Evaluation of a device for attenuation of electron release from dental restorations in a therapeutic radiation field. J Prosthet Dent.

[14] Pan JJ, Zheng BH, Zhang Y, Chen CB, Li JL, Zhang XC (2006). Measurement of setup error in conformal radiotherapy for nasopharyngeal carcinoma. Ai Zheng.

[15] Pham QV, Lavallee AP, Foias A, Roberge D, Mitrou E, Wong P (2018). Radiotherapy immobilization mask molding through the use of 3D-printed head models. Technol Cancer Res Treat.

[16] Liu XQ, Luo W, Lin SR, Liu MZ (2009). Placement repeatability of individual oral stent used in radiotherapy of nasopharyngeal carcinoma. Ai Zheng.

[17] Chen GFFL, San GP, Wang BB, Chen WJ, Jiang F (2015). Analysis of the positioning error of the occluder as the oral stent for head and neck tumor radiotherapy. Zhe Jiang Med J.

[18] Shao HMWX, Yu CH, Zheng YM, Wang JH, Zhou C (2012). Study on the position repeatability of simple and individualized oral stent with cork in radiotherapy of head and neck tumor. Modern Practical Medicine.

[19] Cleland S, Crowe SB, Chan P, Chua B, Dawes J, Kenny L, Lin CY, McDowall WR, Obereigner E, Poroa T, Stewart K, Kairn T (2022). Development of a customisable 3D-printed intra-oral stent for head-and-neck radiotherapy. Tech Innov Patient Support Radiat Oncol.

[20] Liu J, Sun L, Xu W, Wang Q, Yu S, Sun J (2019). Current advances and future perspectives of 3D printing natural-derived biopolymers. Carbohydr Polym.

[21] Al-Rawi SAI, Abouelenein H, Khalil MM, Alabdei HH, Sulaiman AA, Al-Nuaimi DS, Nagdy MEE (2022). Evaluation of conformity and homogeneity indices consistency throughout the course of head and neck cancer treatment with and without using adaptive volumetric modulated arc radiation therapy. Adv Radiat Oncol.

[22] Isobe I, Mori Y, Kaneda N, Hashizume C, Ishiguchi T, Suzuki K (2020). Dosimetric comparison of hypofractionated multi-beam intensity-modulated radiation therapy and volumetric modulated arc therapy with flattened beam and flattening-filter-free beam for skull base meningioma adjacent to optic pathways. Cureus.

[23] Goel A, Tripathi A, Chand P, Singh SV, Pant MC, Nagar A (2010). Use of positioning stents in lingual carcinoma patients subjected to radiotherapy. Int J Prosthodont.

[24] Tino RLM, Yeo A, Kyriakou E, Kron T, Brandt M (2020). Additive manufacturing in radiation oncology: a review of clinical practice, emerging trends and research opportunities. Int J Extrem Manuf.

[25] Singhvi MS, Zinjarde SS, Gokhale DV (2019). Polylactic acid: synthesis and biomedical applications. J Appl Microbiol.

[26] de Albuquerque TL, Marques Junior JE, de Queiroz LP, Ricardo ADS, Rocha MVP (2021). Polylactic acid production from biotechnological routes: a review. Int J Biol Macromol.

[27] Katsura K, Utsunomiya S, Abe E, Sakai H, Kushima N, Tanabe S, Yamada T, Hayakawa T, Yamanoi Y, Kimura S, Wada S, Aoyama H, Hayashi T (2016). A study on a dental device for the prevention of mucosal dose enhancement caused by backscatter radiation from dental alloy during external beam radiotherapy. J Radiat Res.

[28] Bruno JS, Miranda-Silva W, Guedes VDS, Parahyba CJ, Moraes FY, Fregnani ER (2020). Digital workflow for producing oral positioning radiotherapy stents for head and neck cancer. J Prosthodont.

